# Radiosynthesis and Evaluation of ^11^C‑Labeled Imidazolyl Pyrimidine Derivatives for Positron Emission Tomography Imaging of Glycogen Synthase Kinase‑3

**DOI:** 10.1021/acsptsci.5c00032

**Published:** 2025-06-20

**Authors:** Yinlong Li, Kenneth Dahl, Charles S. Elmore, Johan Sandell, Akihiro Takano, Christer Halldin, Lars Farde, Charlotte Ahlgren, Alison Cochrane, Jian Rong, Jiahui Chen, Chunyu Zhao, Xin Zhou, Jimmy S. Patel, Zhendong Song, Ahmad Chaudhary, Yabiao Gao, Zhenkun Sun, Zachary Zhang, Siyan Feng, Achi Haider, Steven H. Liang, Magnus Schou

**Affiliations:** 1 Department of Radiology and Imaging Sciences, 1371 Emory University, 1364 Clifton Road, Atlanta, Georgia 30322, United States; 2 PET Science Centre, Precision Medicine and Biosamples, Oncology R&D, AstraZeneca, Karolinska Institutet, Stockholm 17176, Sweden; 3 Department of Clinical Neuroscience, Centre for Psychiatry Research, Karolinska Institutet and Stockholm County Council, Stockholm 17176, Sweden; 4 Early Chemical Development, Pharmaceutical Sciences, R&D, AstraZeneca Pharmaceuticals, Gothenburg 43183, Sweden; 5 Department of Radiation Oncology, Winship Cancer Institute of Emory University, Atlanta, Georgia 30322, United States; 6 Department of Pharmacology and Chemical Biology, Emory University School of Medicine, Atlanta, Georgia 30322, United States; 7 Department of Biophysics and Radiation Biology, and HUN-REN TKI, Semmelweis University, 1094 Budapest, Hungary

**Keywords:** glycogen synthase kinase-3, imidazolylpyrimidine derivatives, carbon-11, positron emission tomography, radioligand

## Abstract

Glycogen synthase kinase-3 (GSK-3) is a serine/threonine kinase that regulates various biological processes by phosphorylating protein substrates. Dysregulation of GSK-3 is linked to a variety of diseases, including malignancies, diabetes, and neurodegenerative disorders. Moreover, GSK-3 hyperactivity is a potential contributing factor in Alzheimer’s disease, suggesting that GSK-3 inhibition may offer therapeutic benefits. Herein, we report the synthesis and evaluation of five ^11^C-labeled imidazolyl pyrimidine analogues [^11^C]**13a**–**e** (codenamed AZ12646326, AZ12646603, AZ12656261, AZ12977360, and AZ12943203) as novel radioligands for positron emission tomography (PET) imaging of GSK-3. Pharmacological assays showed that compounds **13a**–**e** exhibited high *in vitro* binding affinity to GSK-3β, with *K_i_* values ranging from 2.49 to 4.95 nM. *In vitro* autoradiography confirmed high levels of specific binding in GSK-3-rich regions of the rodent brain, highlighting the promising imaging properties of these analogues. Radiosynthesis of [^11^C]**13a**–**e** was achieved via palladium-promoted carbonylation reactions with [^11^C]carbon monoxide, with excellent radiochemical purity (>99%). However, PET imaging studies in nonhuman primates *in vivo* showed low brain uptake of these radioligands, and [^11^C]**13e** was identified as a *P*-glycoprotein substrate. This study offers valuable insights for optimizing future GSK-3-targeted PET tracers based on the imidazolyl pyrimidine scaffold.

## Graphical abstract

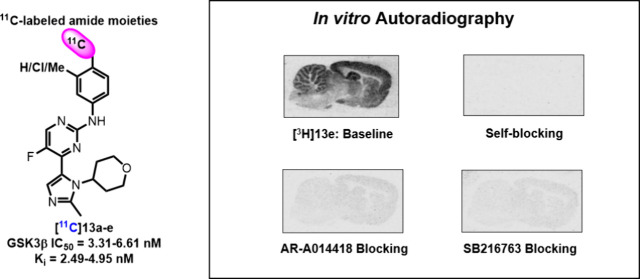

Glycogen synthase kinase-3 (GSK-3) is a serine/threonine protein kinase that plays a crucial role in various cellular processes, such as proliferation, cell signaling, metabolism, and immune responses.^1–3^ GSK-3 comprises two highly homologous isoforms (GSK-3α and GSK-3β) that are highly distributed across various tissues and especially widespread in the central nervous system (CNS).^4^ A growing body of evidence links dysregulation of GSK-3β activity with a variety of pathologies,^5–7^ including Alzheimer’s disease (AD),^8–10^ Parkinson’s disease (PD),^11,12^ type-II diabetes^13–15^ and different malignancies.^16–18^ For example, enhanced activity of GSK-3β prompts hyper-phosphorylation of Tau protein leading to neuronal degeneration, thus contributing to AD pathology.^19–21^ In clinical studies, aberrant GSK-3 activity has been reported in patients with AD, major depressive disorder, or schizophrenia.^22–24^ As such, altered activity may occur without changes in receptor expression, which constitutes a significant challenge for the noninvasive assessment of GSK-3 function. Nonetheless, several studies have reported changes in GSK-3 expression in pathological conditions, such as Huntington’s disease (HD)^25^ and diabetes,^26^ prompting calls for the development of suitable GSK-3-targeted PET radioligands. Along this line, selective inhibition of GSK-3 holds the potential to restore homeostatic GSK-3 function, rendering GSK-3 a promising target for the potential treatment of these diseases.^27,28^ Indeed, lithium is considered the first naturally occurring GSK-3 inhibitor, but its clinical use is hampered by low target selectivity, adverse side effects, and high toxicity.^29,30^

Positron emission tomography (PET) is a molecular imaging technique that enables noninvasively visualizing and quantifying biochemical and physiological functions by tracking the real-time distribution of radioactive tracers.^31–33^ PET imaging of GSK-3 would allow for assessment of its density and to monitor changes in GSK-3 levels within living subjects, thereby enhancing our understanding of GSK-3 related physiology and pathologies.^34,35^ In recent years, several PET radioligands have been reported for PET imaging of GSK-3 (Figure 1). One of the first reported GSK-3-targeted radioligands, [^11^C]**1** (AR-A014418), was not successfully translated due to its low permeability across the blood-brain barrier (BBB).^36^ [^11^C]**2** (PyrATP-1), which was developed from a literature compound (K_i_ = 4.9 nM), revealed poor brain uptake in rodents and nonhuman primates (NHPs) in PET imaging studies.^37^ Similarly, oxadiazole-based probes [^11^C]**3–5** exhibited low brain penetration in mice, despite apparently suitable physicochemical properties.^38^ [^11^C]**6** (SB-216763) was the first GSK-3 radioligand reported to penetrate the BBB in rodents and NHPs. However, [^11^C]**6** displayed a homogeneous uptake across the brain and there was no support for specific binding.^39,40^ The Maleimide-based GSK-3β tracer [^18^F]**7** (IC_50_ = 1.7 nM) demonstrated moderate brain uptake, although no saturable binding was observed in rodents.^41^ [^11^C]**8** (PF-04082367) demonstrated promising brain uptake and binding specificity in NHPs, but further kinetic modeling analysis is needed for high species translation.^42^ [^11^C]**9** (A1070722) was identified as a selective GSK-3 ligand (K_i_ = 0.6 nM) in the NHP brain, but it lacked sufficient brain exposure.^43^ A series of isonicotinamide derivatives, including [^11^C]**10** (CMP),^44^ [^18^F]**11** ^45^ and [^18^F]**12** ^46,47^ labeled with either carbon-11 or fluorine-18 were recently reported, but they showed insignificant uptake in the rodent brain and/or low-to-moderate specific binding.

**Figure 1:**
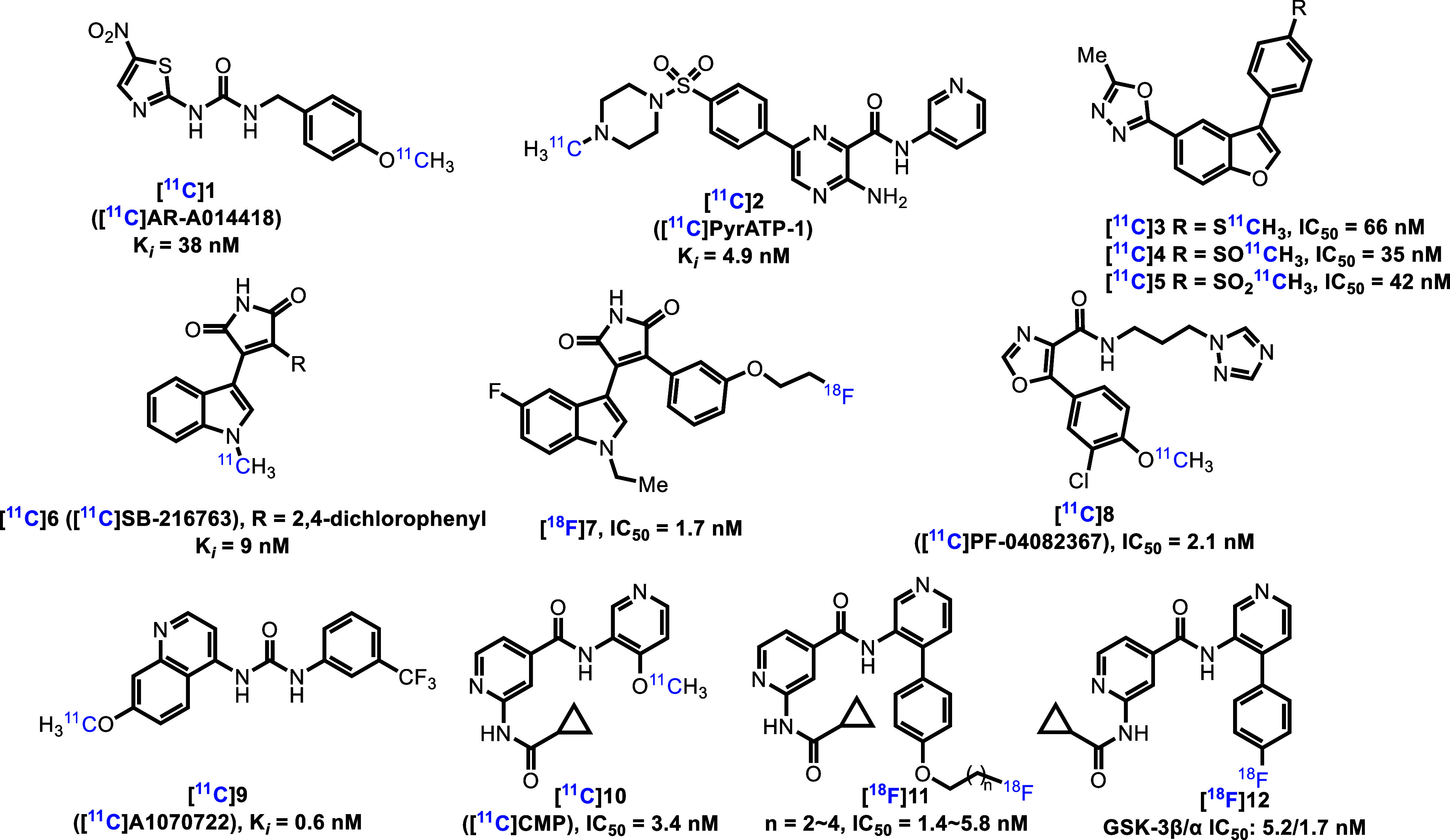
Potential GSK-3 PET ligands.

Despite the above-mentioned attempts, a clinically validated GSK-3 radioligand is currently lacking. As part of our continuous efforts on GSK-3-targeted PET tracer development,^42,47^ we focused on imidazolyl pyrimidine derivatives guided by established structure–activity relationship (SAR) data, which highlight their potency and selectivity as GSK-3 inhibitors.^48,49^ The imidazole ring serves as a critical pharmacophore, facilitating essential hydrogen bonding and π-π stacking interactions within the ATP-binding site of GSK-3. Similarly, the pyrimidine scaffold is a privileged structure in kinase inhibitor design by enhancing binding affinity and selectivity. Herein, we describe the radiolabeling and preclinical evaluation of a series of imidazolyl pyrimidine derivatives, [^11^C]**13a**–**e**, as potential GSK-3 PET ligands (Figure 2).

**Figure 2:**
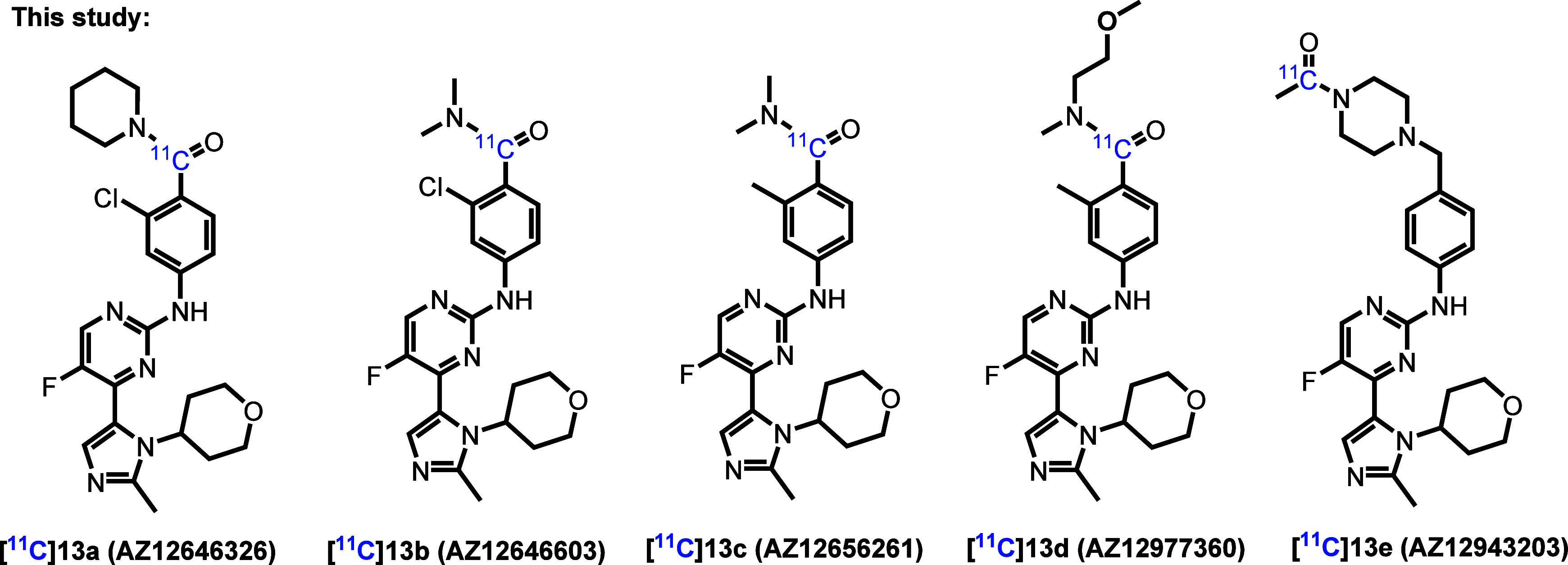
Chemical structures of novel GSK-3 PET ligands.

## Result and Discussion

The pharmacology and physicochemical properties of compounds **13a**–**13e** are depicted in Table 1. To evaluate binding affinities, saturation binding assays using tritiated ligands [^3^H]**13a**, [^3^H]**13c**, [^3^H]**13d**, [^3^H]**13e** (Scheme S1) were performed using the hippocampal tissue of Sprague–Dawley (SD) rat brains. As depicted in Table 1A, total binding (TB), nonspecific binding (NSB), and specific binding (SB) of the respective tritiated ligands were measured with increasing concentrations of the radioligands. The dissociation constant (K_d_) and capacity (*B*_max_) of each radioligand were calculated to confirm their binding affinity.

**Table 1:**
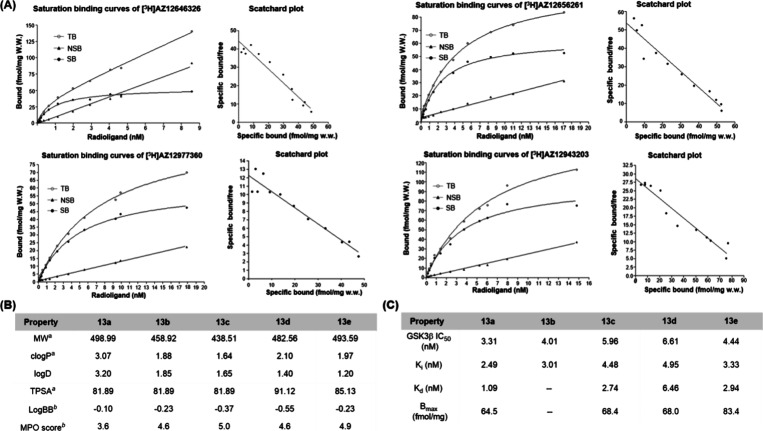
(A) Representative Saturation Binding Curves and Scatchard Plots for Tritiated Ligands [^3^H]**13a**, [^3^H]**13c**, [^3^H]**13d**, and [^3^H]**13e** in the Hippocampus of SD Rat Brains; In Silico (B) Physicochemical and (C) Pharmacology Properties of **13a**–**e**

a Values were calculated with ChemDraw 21.0 software.

b Values were predicted with ACD/laboratories. [^3^H]**13b** was not subjected to a saturation binding experiment; therefore, *K_d_* and *B*_max_ values are not provided.

All the tested radioligands demonstrated high binding affinity to GSK-3, with K_d_ values ranging from 1.09 nM to 6.46 nM (Table 1C). The enzyme density, *B*_max_, quantified by each radioligand ranged from 64.5 to 83.4 fmol/mg. Notably, all *B*_max_/K_d_ ratios were higher than 10, which is considered favorable for *in vivo* PET neuroimaging.^50^ Similarly, in silico physicochemical properties of **13a**–**13e** such as molecular weight (MW< 500 g/mol), lipophilicity (clogP: 1.64–3.07 and LogD: 1.20–3.20) and topological polar surface area (TPSA: 81.89–91.12) indicated favorable brain permeability (Table 1B).^51^

Multiparameter optimization (MPO) and brain/plasma concentration at steady-state (logBB) are widely used to predict BBB permeability. A desirability score of ≥ 4.0 for MPO and ≥ – 1 for logBB are generally considered thresholds for potential BBB penetration.^52,53^ As shown in Table 1B, compounds **13a**–**13e** met these thresholds, suggesting their potential to cross the BBB. Additionally, compounds **13a**–**13e** exhibited high inhibitory potency toward GSK-3β, with low IC_50_ (3.31–6.61 nM) and K_i_ (2.49–4.95 nM) values (Table 1C). A detailed ADME/PK (absorption, distribution, metabolism, excretion and pharmacokinetics) profiling was conducted, and the results are listed in Table 2. The apparent permeability coefficient (P_app_ A–B) of **13b**–**13e** was measured as 12.7–22.7 × 10^–6^ cm/s, along with the efflux ratio (P_app_ B–A/P_app_ A–B) ranging from 0.7 to 2.1. These values meet the standards that BBB permeability is considered high if P_app_ A–B is ≥ 3.0 × 10^–6^ cm/s and the efflux ratio is <3.0.^54^ All brain-to-plasma unbound fraction ratios (*f*_u_ brain) > 1, indicating that a greater proportion of the compound could remain unbound (free) in the brain and available for pharmacological effect. The solubility of **13a**–**13e** was also measured to be greater than 150 μM in DMSO.

**Table 2:** ADME/PK Analysis of Compounds **13a**–**13e**

**Property**	**13a**	**13b**	**13c**	**13d**	**13e**
*P*_app_ A-B (× 10^–6^ cm/s)	--	22.7	16.2	21.9	12.7
efflux ratio	--	1.2	2.1	0.7	1.4
*f_u_* brain (%)	3.6	13	19	26	12
solubility (μM)^a^	234	155	>400	--	362

**Property**	**13a**	**13b**	**13d**	**13e**	**13f**
mean Prot binding (% free)	--	5.6	11	11.1	7.6
median CLint (μ/min/10^6^ cells)	34.2	17.5	15.4	12	11
mean *t*_1/2_ (h)	--	2.32	3.65	1.73	2.27
mean bioavailability (%)	--	39.8	32.9	10.1	30.9
CYP1A2 IC_50_ (μM)	>2	>2	>2	--	>2
CYP2C9 IC_50_ (μM)	>2	>2	>2	--	>2
CYP2D6 IC_50_ (μM)	>2	>2	>2	--	>2
CYP3A4 IC_50_ (μM)	>2	>2	>2	--	>2
hERG IC_50_ (μM)	4.52	18.8	>33	20.5	>33

a Solubility in dry DMSO at pH = 7.4.

The pharmacokinetics assays including mean protein binding, median intrinsic clearance (CLint), mean half-life (t_1/2_) and mean bioavailability revealed that these compounds exhibit reasonable clearance and good bioavailability. The inhibitory properties of **13a**–**13e** on four cytochrome P450 isoforms (CYP1A2, CYP2C9, CYP2D6 and CYP3A4) were greater than 2 μM. On the other hand, no hERG channel inhibition was observed (IC_50_ ≥ 4.52 μM), indicating no cardiac safety concerns. Therefore, all pharmacology and ADME assays suggested that compounds **13a**–**13e** may be considered as potential radioligands with brain penetration and high affinity for GSK-3.

In the next step, we performed *in vitro* autoradiography studies on rat brain tissue sections to investigate the binding selectivity and distribution of compounds [^3^H]**13a**–**13e** (Figure 3). The results demonstrated that [^3^H]**13a**–**13e** exhibited heterogeneous distributions. Among these, [^3^H]**13d** displayed the lowest level of radioactive accumulation, while [^3^H]**13e** exhibited the highest and most regionally distinct uptake. Notably, [^3^H]**13e** showed high binding in the cortex and hippocampus, moderate uptake in the striatum and cerebellum, and relatively low binding in the thalamus. This distribution pattern closely correlates with previously reported GSK-3 expression profiles from immunohistochemistry and in situ hybridization studies.^55,56^ Co-incubation with cold reference and validated GSK-3β inhibitors (AR-A014418 and SB-216763) significantly reduced the radioactive signal in all the brain regions, confirming specific binding to GSK-3β. These findings support the potential of this compound series for further development as GSK-3β-targeted neuroimaging probes.

**Figure 3:**
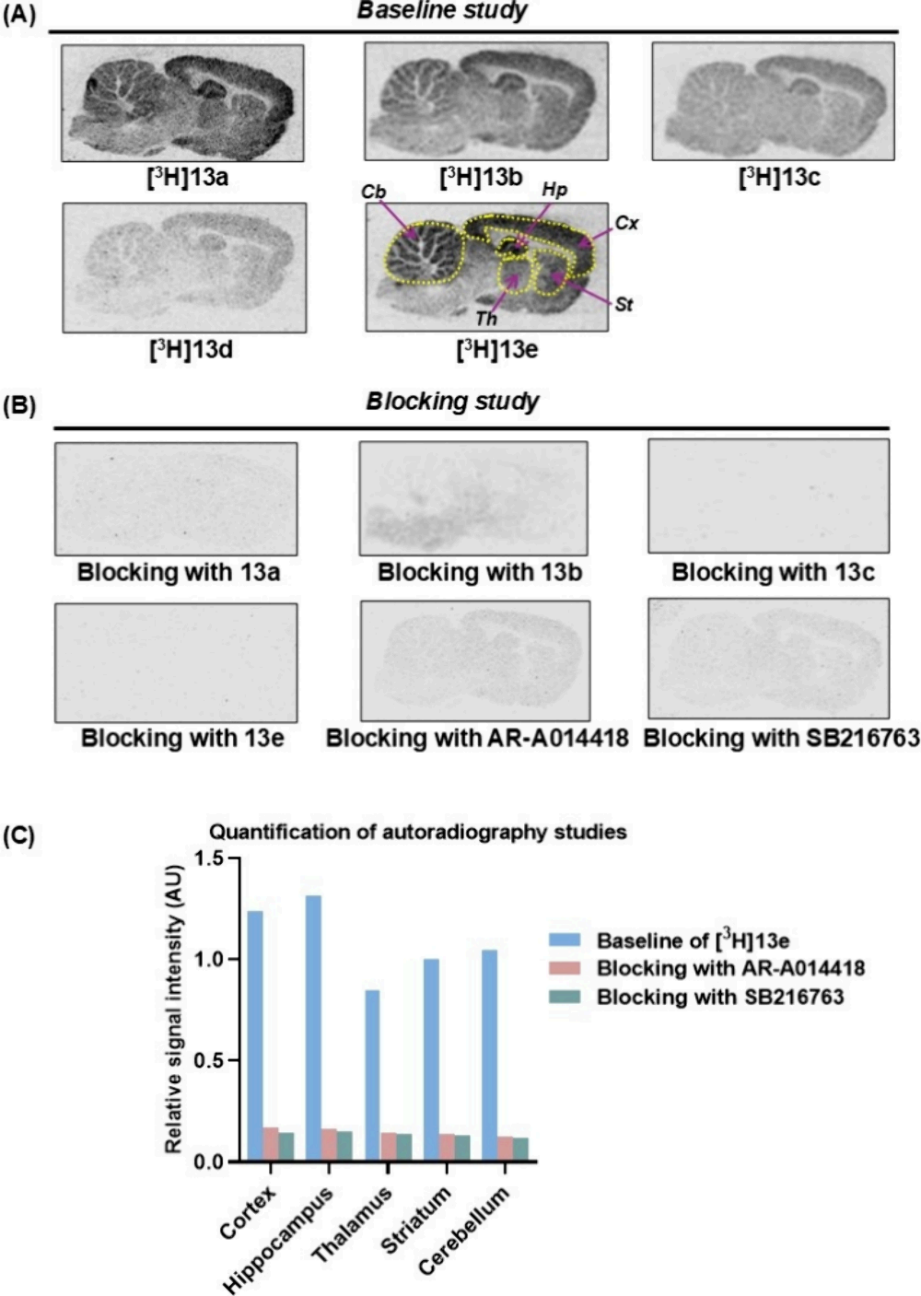
Representative *in vitro* autoradiograph of [^3^H]**13a**, [^3^H]**13b**, [^3^H]**13c**, [^3^H]**13d**, and [^3^H]**13e** under baseline (A) and blocking (B) conditions (blocker = 3 or 5 μM). (C) Quantitative analysis of autoradiograms with [^3^H]**13e**.

To further evaluate the *in vivo* property of compounds **13a**–**13e**, we designed precursors **14–16** for radiolabeling with PET radionuclide carbon-11 (half-life = 20.4 min). As shown in Scheme 1 **a-c**, the radiosynthesis of [^11^C]**13a–d** was achieved through palladium-mediated carbonylation reactions with [^11^C]carbon monoxide ([^11^C]­CO) and aryl iodides **14** and **15**. In contrast, [^11^C]**13e** was synthesized via the coupling of [^11^C]­CO with iodomethane (MeI) and the amine precursor **16** (Scheme 1d). The use of MeI represents a distinct reaction pathway compared to the aryl iodides used for [^11^C]**13a–d**. In particular, the radiolabeling approach was enabled by a key N-acylation step on the piperazine moiety, which provided a suitable precursor for efficient [^11^C]­methylation using MeI. The resulting radioligands were produced in 1.6%–13.8% radiochemical yields (RCY, decay-corrected) with excellent radiochemical purities (RCP > 99%) and molar activities (Am = 22–99 GBq/μmol) at the end of synthesis (EOS).

**Scheme 1:**
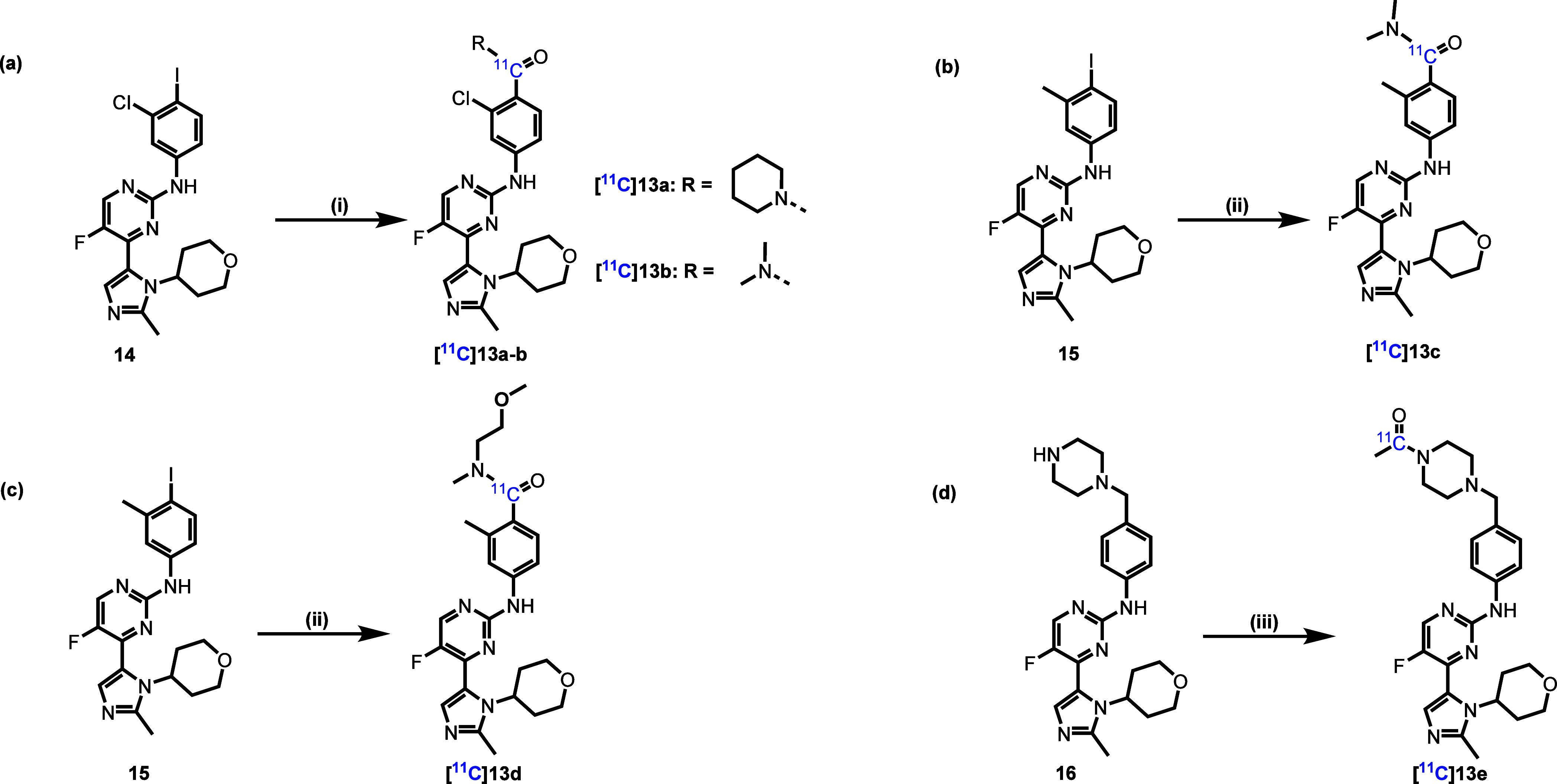
Radiosynthesis of (a) **[** ^**11**^ **C]­13a–b**, (b) **[** ^**11**^ **C]­13c**, (c) **[** ^**11**^ **C]­13d**, and (d) **[** ^**11**^ **C]­13e** ^a^

a (i) [^11^C]­CO, palladium­(π-cinnamyl) chloride dimer, xantphos or Pd­(PPh_3_)_4_, piperidine or diethylamine, THF, 100 °C, 5 min. (ii) [^11^C]­CO, palladium­(π-cinnamyl) chloride dimer, xantphos, diethylamine, or (2-methoxyl)­methylamine, THF, 100 °C, 5 min. (iii) [^11^C]­CO, Pd­(PPh_3_)_4_, MeI, THF, 100 °C, 5 min

Then, we sought to assess the *in vivo* PET imaging performance characteristics of [^11^C]**13a**–**e** in NHPs, and the results are presented in Figure 4. To our surprise, none of the radioligands exhibited significant brain uptake and radioactivity in the brain cleared rapidly at 1–2 min postinjection. Time–activity curves (TACs) expressed in standard uptake value (SUV) indicated that [^11^C]**13e** exhibited relatively higher signals in the brain, although potential spillover effects cannot be excluded in dynamic PET scans (0–120 min). In addition, all five radioligands displayed a heterogeneous distribution pattern across brain regions (Figure S2). Despite favorable in silico predictions for CNS penetration, the low brain uptake observed in NHPs suggests additional factors may be limiting BBB permeability. One potential contributor is metabolic instability of those tracers may reduce the amount of intact tracers to cross the BBB. Another important consideration is efflux by ATP-binding cassette (ABC) transporters, such as P-glycoprotein (P-gp) that is highly expressed at the BBB and can actively pump substrates out of the CNS. To investigate whether **13a**–**e** are P-gp efflux substrates, a P-gp inhibition study was conducted. As shown in Figures 5A and 5 **B**, inhibiting P-gp with Tariquidar (6 mg/kg) significantly increased the brain uptake of [^11^C]**13e**. Brain regional distribution volume (V_T_) values for [^11^C]**13e** increased by 138%–313% compared to the respective control studies, indicating that these radioligands may be P-gp substrates. Additionally, although the imidazolyl-pyrimidine scaffold generally exhibits physicochemical properties compatible with CNS penetration, specific structural features such as the presence of hydrogen bond donors, high tPSA, or ionizable groups may contribute to poor passive diffusion across the BBB. To enhance brain uptake in future analogs, various medicinal chemistry strategies could be employed, such as hydrogen bond donor/acceptor optimization, fine-tuning lipophilicity, steric shielding of efflux motifs, and bioisosteric replacements. A potential limitation of this study is the lack of blood sampling during the PET scans, which precluded assessment of [^11^C]**13e** plasma concentrations following tariquidar pretreatment. While the tariquidar protocol is a widely used standard to probe P-gp–mediated efflux in PET tracer development, alternative approaches such as studies in P-gp knockout animals may offer additional insight. However, genetic deletion of P-gp has been associated with broader alterations in BBB integrity, which may also confound interpretation of tracer distribution.

**Figure 4:**
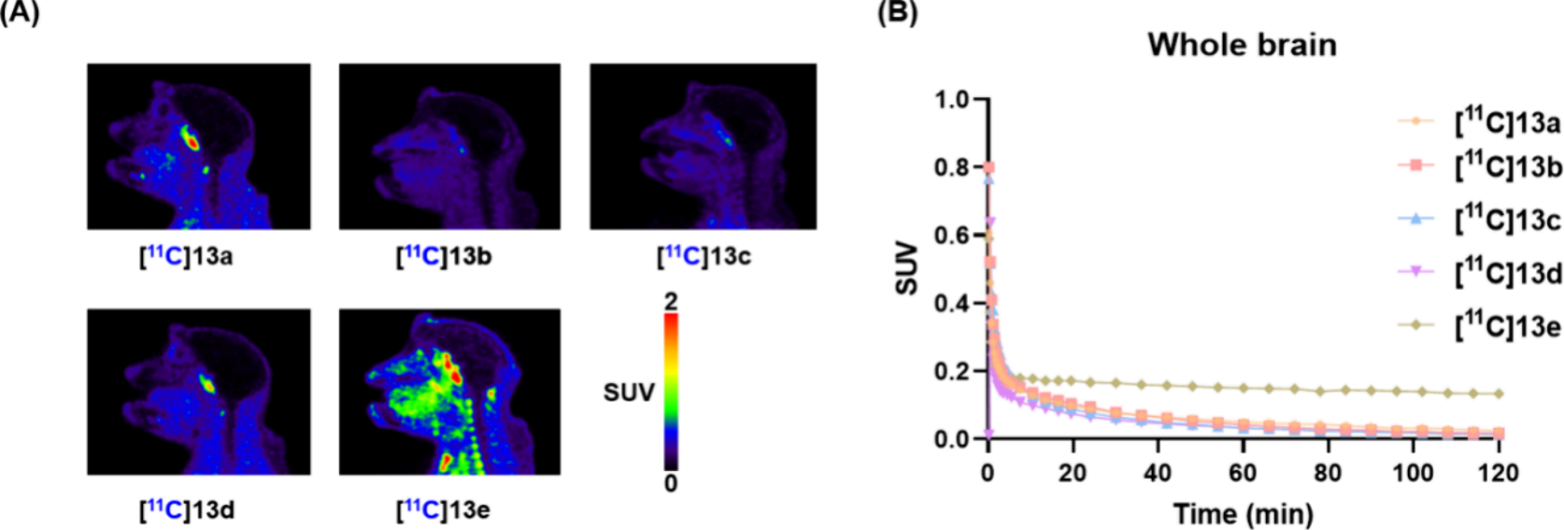
(A) Summed (0 to 120 min) PET images and (B) TACs of [^11^C]**13a**, [^11^C]**13b**, [^11^C]**13c**, [^11^C]**13d**, and [^11^C]**13e** in NHP.

**Figure 5:**
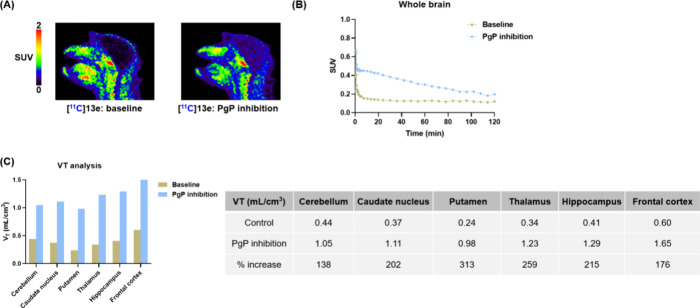
PET imaging study of PgP inhibition effects (pretreatment with 6 mg/kg tariquidar) on (A) radioactivity levels of [^11^C]**13e** and (B) TACs and (C) VT values in NHP brains.

## Conclusions

In summary, we identified a series of imidazolyl pyrimidine analogues **[^11^C]­13a**–**e** as potential radioligands for PET imaging of GSK-3. Pharmacological assays demonstrated that compounds **13a**–**e** possess high *in vitro* specific binding affinity with nanomolar IC_50_ and *K*_i_ values. Autoradiography studies using tritium-labeled radioligands on rodent brain tissue sections demonstrated high specific binding to brain regions known to highly express GSK-3. Co-incubation with cold-validated GSK-3β inhibitors (AR-A014418 and SB-216763) confirmed specific binding to GSK-3β. The radiosynthesis was accomplished through palladium-promoted carbonylation reactions with [^11^C]­CO, yielding [^11^C]**13a**–**e** in excellent radiochemical purity and high molar activity. Although *in vivo* PET imaging in the NHP brain did not exhibit sufficient brain uptake, insights from this study suggest the limitation of the current chemical scaffold and improved brain permeability is necessary for developing future GSK-3 targeted PET tracers.

## Experimental Section

### Materials and Methods

The imidazolyl pyrimidine-based analogues **13a**–**13e** and corresponding precursors were synthesized following the procedure described in our patent.^57^ High-performance liquid chromatography (HPLC) solvents were sourced from Fisher (Sweden). Unless specified otherwise, all additional reagents and solvents were provided by Sigma-Aldrich (Sweden) and used as received. Analytical HPLC was conducted with a Hitachi L-6200 gradient pump (Tokyo, Japan) equipped with a variable wavelength UV detector (λ = 254 nm) and a Bioscan β^+^ flow detector. A reverse phase column (Zorbax eclipse, 5 μm, 3.9 × 150 mm) with an eluent of acetonitrile/0.1 *M* ammonium formate (gradient method, 10–90% CH_3_CN in 7 min) at a flow rate of 3 mL/min was used. All radioligands were confirmed by coinjection with the corresponding cold compounds.

### Radiochemistry

No-carrier-added [^11^C]­CO_2_ (approximately 50 GBq) was generated through the ^14^N­(p,α)^11^C nuclear reaction, by irradiating a PETtrace 800 cyclotron (GE, Uppsala, Sweden) target with a mixture of nitrogen and 1% oxygen using a 16.4 MeV proton beam (35 μA, 30 min). The produced [^11^C]­CO_2_ was then converted to [^11^C]­CO using a preheated quartz glass column (6 × 4 × 180 mm; outer diameter × inner diameter × length) heated in a Carbolite oven 850 °C and packed with molybdenum powder (1.5 g, < 150 μm, 99.99% trace metals basis, Sigma-Aldrich, Sweden). [^11^C]­CO was subsequently treated with coupling reagents (halide precursor, Pd-source, supporting ligand, and amine precursor) in anhydrous THF using a high-pressure microautoclave system (GE, Uppsala, Sweden) following a previously established method.^58^ For specific reaction details, e.g., reagent amounts, Pd-catalyst, and supporting ligand, see the following sections below. Purification and formulation were carried out with a computer-controlled automated system (DM Automation, Sweden). Semipreparative HPLC was equipped with a reversed-phase C-18 column (μBondapak, 10 μm, 10 × 300 mm, Waters) with a UV detector wavelength of 254 nm in line with a radiation detector. The purified radioligands underwent further purification via solid phase extraction (SepPak, C18 plus short, Waters) and were then formulated in a solution of 10% ethanol in phosphate-buffered saline (PBS, pH 7.4).

### Radioligand Saturation Binding Assay

For membrane preparation, hippocampal tissue from adult rats (Sprague–Dawley) was dissected out, weighed, and frozen in 0.32 *M* sucrose at −70 °C. On the experiment day, the tissue was thawed, homogenized in 50 mM Tris, 5 mM EDTA (pH 7.4) and then centrifuged at 48 000 xg for 15 min at +4 °C. The pellet was diluted in binding buffer (50 mM Tris, 4 mM MgCl_2_, 1 mM EDTA pH 7.4) to a final concentration of 2.5 mg w.w./mL. Saturation binding experiments were performed in duplicate, with each tube containing 1 mg w.w. in 0.5 mL binding buffer and 0.1 – 17 nM (11–12 concentrations) of radioligand. Nonspecific binding was measured by adding 10 μM AR-A014418. The assays were incubated for 2–3 h at room temperature and terminated by quick filtration using Whatman GF/B filters pretreated with 0.3% polyethylenimine on a Brandel cell harvester. Radioactivity was measured with a Packard Tri-Carb 2900TR liquid scintillation counter, and data were processed by nonlinear regression analyses using PRISM 4.03.

### *In Vitro* Binding Assays

The inhibition of GSK3β activity is assessed using a scintillation proximity assay (SPA) in a 96-well microtiter plate format. The assay utilizes purified human recombinant GSK3β enzyme (∼1 nM active), [γ-^33^P]­ATP (0.04 μCi), and a biotinylated peptide substrate (Biotin-AAEELDSRAGS­(PO_3_H_2_)­PQL) at a final concentration of 2 μM. Reactions are carried out in an assay buffer containing MOPS, EDTA, β-mercaptoethanol, Brij 35, glycerol, and BSA, and preincubated for 10–15 min at room temperature. Inhibitor testing is conducted in duplicate using 10 different concentrations. The kinase reaction is initiated by adding ATP (1 μM final) in the presence of 50 mM Mg­(Ac)_2_. After a 15 min incubation at room temperature, the reaction is terminated by adding a solution (25 μL) containing 5 mM EDTA, 50 μM ATP, 0.1% Triton X-100, and 10 mg/mL streptavidin-coated SPA beads, followed by the addition of distilled water (50 μL). After a 6-h incubation, bound radioactivity is measured using a MicroBeta Trilux liquid scintillation counter (1450 MicroBeta Trilux, Wallac). Data are analyzed using nonlinear regression with variable slope (GraphPad Prism or Excel Fit), and results are reported as K_i_ values calculated via the Cheng-Prusoff equation.

### *In Vitro* Autoradiography

*In vitro* autoradiography was conducted on 10 μm thick cry-cut tissue sections of rat tissue that had been thaw-mounted onto microscope slides. The brain sections were incubated in 50 mM Tris-HCl buffer at room temperature and then incubated with [^3^H]­ligand for 30 min. The sections were rinsed three times for 10 min each in Tris buffer at 1 °C, followed by a quick rinse in deionized water at 1 °C, and then air-dried at room temperature with a fan. The radiolabeled sections and Amersham plastic tritium standards were then exposed to phosphoimager plates (Fuji BAS-TR2040) overnight. Blocking studies were conducted using a 1000-fold excess of the cold reference compounds, and validated GSK-3β inhibitors AR-A014418 and SB-216763. These inhibitors were selected based on their well-established selectivity and potency for GSK-3β, as reported in prior pharmacological and imaging studies.^39,59^ AR-A014418 and SB-216763 were applied at 3 μM, significantly exceeding the IC_50_ values (4.44 nM) of **13e**, to ensure complete displacement of specific radioligand binding. This micromolar concentration is consistent with established methods for GSK-3 radioligand autoradiography blocking experiments.^60^

### Radiosynthesis of [^11^C]**13a–e**

Palladium­(π-cinnamyl)­chloride dimer (1.4 mg), xantphos (3.4 mg), and halide precursor (2 mg) were added to a 4 mL vial. After flushing the vial with nitrogen, piperidine (5 μL) and 600 μL anhydrous THF was added. The reaction mixture was then loaded into the reagent loop of the module, from which it was directed into the microautoclave precharged [^11^C]­CO and subsequently reacted at 100 °C for 5 min. [^11^C]**13a** was purified at a flow rate of 6 mL/min with acetonitrile/0.1*M* ammonium formate (v/v= 40/60) as the eluting solvent (t_R_ = 10 min). [^11^C]**13a** was reformulated with SPE (SepPak, C18 plus short, Waters) in a mixture of phosphate-buffered saline (10 mL, pH 7.4) and 1 mL EtOH. The resulting solution was sterilized by sterile filtration. Pure [^11^C]**13a** was synthesized in 4% RCY (decay-corrected) at EOS (RCP > 99%, Am = 62 GBq/μmol). Following a similar procedure, [^11^C]**13b**–**d** were synthesized in 1.6%–13.8% RCY (decay-corrected) with excellent radiochemical purities (RCP > 99%) and molar activities (Am = 22–99 GBq/μmol) at EOS.

### [^11^C]**13e** Was Synthesized According to the Following Procedure

Tetrakis­(triphenylphosphine)­palladium­(0) (3.0 mg) and methyl iodide (20 μL) were placed in an oven-dried 4 mL vial. The vial was flushed with nitrogen gas before amine precursor **16** (3 mg) and 600 μL anhydrous THF was added. The resulting mixture was loaded into the reagent loop of the module from where it was transferred into the microautoclave precharged with [^11^C]­CO. The mixture was heated at 100 °C for 5 min. [^11^C]**13e** was purified with 30:70 acetonitrile/0.1% TEA as mobile phase at a flow rate of 6 mL/min (t_R_ = 16 min). The purified products were reformulated using SPE (SepPak, C18 plus short, Waters) in a mixture of phosphate-buffered saline (10 mL, pH 7.4) and 1 mL ethanol. The resulting solution was sterilized by sterile filtration. Pure [^11^C]**13e** was synthesized in 12.9% RCY (decay-corrected) at EOS (RCP > 99%, Am = 24 GBq/μmol).

### NHP PET Imaging

PET experiments were conducted on anesthetized healthy cynomolgus monkeys, following approval from the Stockholm Animal Research Ethical Committee (Dnr. 4820/06–600). Anesthesia was induced with an intramuscular injection of ketamine hydrochloride (∼10 mg/kg, Ketalar, Pfizer) and maintained via a mixture of sevoflurane (2–8%, Abbott Scandinavia AB), oxygen (∼40%), and medical air after endotracheal intubation. The monkeys’ heads were stabilized with a fixation device, and body temperature was regulated with a Bair Hugger model 505 (Arizant Healthcare, MN, USA) and continuously monitored using an esophageal thermometer. Each radioligand was administered ([^11^C]**13a** = 149 MBq; [^11^C]**13b** = 155 MBq; [^11^C]**13c** = 164 MBq; [^11^C]**13d** = 144 MBq; [^11^C]**13e** = 156 MBq) as an intravenous bolus injection and dynamic PET images were acquired for 125 min using the high-resolution research tomograph (HRRT; Siemens Molecular Imaging, Knoxville, TN, USA).

## Supplementary Material



## Notes

Syntheses of [^3^H]**13a**–**e** (Scheme S1) and TACs of [^11^C]**13a**, [^11^C]**13b**, [^11^C]**13c**, [^11^C]**13d**, and [^11^C]**13e** in various NHP brain regions (Figure S1) (PDF)

## References

[1] Woodgett J. R. (1990). Molecular
cloning and expression of glycogen synthase kinase-3/factor A. EMBO Journal.

[2] Beurel E., Grieco S. F., Jope R. S. (2015). Glycogen
synthase kinase-3 (GSK3):
Regulation, actions, and diseases. Pharmacology
& Therapeutics.

[3] Patel, P. ; Woodgett, J. R. , Chapter Eight - Glycogen Synthase Kinase 3: A Kinase for All Pathways? Current Topics in Developmental Biology, Jenny, A. , Ed. Academic Press: 2017, 123, 277–302, DOI: 10.1016/bs.ctdb.2016.11.011. 28236969

[4] Laura
Sayas C., Jurado J., Avila J., Villanueva N. (2012). Structural
and Functional Relationships Between GSK3α and GSK3β Proteins. Current Biotechnology.

[5] Duda P., Wiśniewski J., Wójtowicz T., Wójcicka O., Jaśkiewicz M., Drulis-Fajdasz D., Rakus D., McCubrey J. A., Gizak A. (2018). Targeting
GSK3 signaling as a potential therapy of neurodegenerative
diseases and aging. Expert Opinion on Therapeutic
Targets.

[6] Wang L., Li J., Di L.-j. (2022). Glycogen synthesis
and beyond, a comprehensive review
of GSK3 as a key regulator of metabolic pathways and a therapeutic
target for treating metabolic diseases. Medicinal
Research Reviews.

[7] Jope R. S., Yuskaitis C. J., Beurel E. (2007). Glycogen Synthase Kinase-3 (GSK3):
Inflammation, Diseases, and Therapeutics. Neurochem.
Res..

[8] Hooper C., Killick R., Lovestone S. (2008). The GSK3 hypothesis
of Alzheimer’s
disease. Journal of Neurochemistry.

[9] Takashima A. (2006). GSK-3 is essential
in the pathogenesis of Alzheimer’s disease. J. Alzheimer’s Disease.

[10] Sayas C. L., Ávila J. (2021). GSK-3 and Tau: A Key Duet in Alzheimer’s Disease. Cells.

[11] Li J., Ma S., Chen J., Hu K., Li Y., Zhang Z., Su Z., Woodgett J. R., Li M., Huang Q. (2020). GSK-3β Contributes
to Parkinsonian Dopaminergic Neuron Death: Evidence From Conditional
Knockout Mice and Tideglusib. Front. Mol. Neurosci..

[12] Golpich M., Amini E., Hemmati F., Ibrahim N. M., Rahmani B., Mohamed Z., Raymond A. A., Dargahi L., Ghasemi R., Ahmadiani A. (2015). Glycogen synthase
kinase-3 beta (GSK-3β) signaling:
Implications for Parkinson’s disease. Pharmacol. Res..

[13] Gao C., Hölscher C., Liu Y., Li L. (2012). GSK3: a key
target
for the development of novel treatments for type 2 diabetes mellitus
and Alzheimer disease. Rev. Neurosci.

[14] MacAulay K., Woodgett J. R. (2008). Targeting glycogen
synthase kinase-3 (GSK-3) in the
treatment of Type 2 diabetes. Expert Opinion
on Therapeutic Targets.

[15] Wagman A. S., Johnson K. W., Bussiere D. E. (2004). Discovery
and Development of GSK3
Inhibitors for the Treatment of Type 2 Diabetes. Curr. Pharm. Des..

[16] Remsing
Rix L. L., Kuenzi B. M., Luo Y., Remily-Wood E., Kinose F., Wright G., Li J., Koomen J. M., Haura E. B., Lawrence H. R., Rix U. (2014). GSK3 Alpha and Beta
Are New Functionally Relevant Targets of Tivantinib in Lung Cancer
Cells. ACS Chem. Biol..

[17] Mancinelli R., Carpino G., Petrungaro S., Mammola C. L., Tomaipitinca L., Filippini A., Facchiano A., Ziparo E., Giampietri C. (2017). Multifaceted
Roles of GSK-3 in Cancer and Autophagy-Related Diseases. Oxidative Medicine and Cellular Longevity.

[18] McCubrey J. A., Steelman L. S., Bertrand F. E., Davis N. M., Sokolosky M., Abrams S. L., Montalto G., D’Assoro A. B., Libra M., Nicoletti F., Maestro R., Basecke J., Rakus D., Gizak A., Demidenko Z., Cocco L., Martelli A. M., Cervello M. (2014). GSK-3 as potential
target for therapeutic intervention in cancer. Oncotarget.

[19] Hanger D. P., Hughes K., Woodgett J. R., Brion J.-P., Anderton B. H. (1992). Glycogen
synthase kinase-3 induces Alzheimer’s disease-like phosphorylation
of tau: Generation of paired helical filament epitopes and neuronal
localisation of the kinase. Neurosci. Lett..

[20] Timm T., Balusamy K., Li X., Biernat J., Mandelkow E., Mandelkow E.-M. (2008). Glycogen Synthase Kinase (GSK) 3β; Directly Phosphorylates
Serine 212 in the Regulatory Loop and Inhibits Microtubule Affinity-regulating
Kinase (MARK) 2. J. Biol. Chem..

[21] Sturchler-Pierrat C., Abramowski D., Duke M., Wiederhold K.-H., Mistl C., Rothacher S., Ledermann B., Bürki K., Frey P., Paganetti P. A., Waridel C., Calhoun M. E., Jucker M., Probst A., Staufenbiel M., Sommer B. (1997). Two amyloid precursor protein transgenic
mouse models with Alzheimer disease-like pathology. Proc. Natl. Acad. Sci. U. S. A..

[22] Kozlovsky N., Belmaker R. H., Agam G. (2002). GSK-3 and
the neurodevelopmental
hypothesis of schizophrenia. European Neuropsychopharmacology.

[23] Lovestone S., Killick R., Di Forti M., Murray R. (2007). Schizophrenia as a
GSK-3 dysregulation disorder. Trends in Neurosciences.

[24] Forlenza O. V., Torres C. A., Talib L. L., de Paula V. J., Joaquim H. P. G., Diniz B. S., Gattaz W. F. (2011). Increased platelet GSK3B activity
in patients with mild cognitive impairment and Alzheimer’s
disease. Journal of Psychiatric Research.

[25] L’Episcopo F., Drouin-Ouellet J., Tirolo C., Pulvirenti A., Giugno R., Testa N., Caniglia S., Serapide M. F., Cisbani G., Barker R. A., Cicchetti F., Marchetti B. (2016). GSK-3β-induced Tau pathology drives hippocampal
neuronal cell death in Huntington’s disease: involvement of
astrocyte–neuron interactions. Cell Death
& Disease.

[26] Nikoulina S. E., Ciaraldi T. P., Mudaliar S., Mohideen P., Carter L., Henry R. R. (2000). Potential role of glycogen synthase kinase-3 in skeletal
muscle insulin resistance of type 2 diabetes. Diabetes.

[27] Cohen P., Goedert M. (2004). GSK3 inhibitors: development and
therapeutic potential. Nat. Rev. Drug Discovery.

[28] Arfeen M., Bharatam V. P. (2013). Design of Glycogen
Synthase Kinase-3 Inhibitors: An
Overview on Recent Advancements. Curr. Pharm.
Des..

[29] Klein P. S., Melton D. A. (1996). A molecular mechanism for the effect
of lithium on
development. Proc. Natl. Acad. Sci. U. S. A..

[30] Stambolic V., Ruel L., Woodgett J. R. (1996). Lithium inhibits glycogen synthase
kinase-3 activity and mimics Wingless signalling in intact cells. Curr. Biol..

[31] Rong J., Haider A., Jeppesen T. E., Josephson L., Liang S. H. (2023). Radiochemistry for positron emission
tomography. Nat. Commun..

[32] Deng X., Rong J., Wang L., Vasdev N., Zhang L., Josephson L., Liang S. H. (2019). Chemistry for Positron Emission Tomography:
Recent Advances in 11C-, 18F-, 13N-, and 15O-Labeling Reactions. Angew. Chem., Int. Ed..

[33] Krishnan H. S., Ma L., Vasdev N., Liang S. H. (2017). 18F-Labeling of Sensitive Biomolecules
for Positron Emission Tomography. Chem. - Eur.
J..

[34] Pandey M. K., DeGrado T. R. (2016). Glycogen Synthase
Kinase-3 (GSK-3)-Targeted Therapy
and Imaging. Theranostics.

[35] Narayanaswami V., Dahl K., Bernard-Gauthier V., Josephson L., Cumming P., Vasdev N. (2018). Emerging PET Radiotracers
and Targets
for Imaging of Neuroinflammation in Neurodegenerative Diseases: Outlook
Beyond TSPO. Molecular Imaging.

[36] Vasdev N., Garcia A., Stableford W. T., Young A. B., Meyer J. H., Houle S., Wilson A. A. (2005). Synthesis
and ex vivo evaluation
of carbon-11 labelled N-(4-methoxybenzyl)-N′-(5-nitro-1,3-thiazol-2-yl)­urea
([^11^C]­AR-A014418): A radiolabelled glycogen synthase kinase-3β
specific inhibitor for PET studies. Bioorg.
Med. Chem. Lett..

[37] Cole E. L., Shao X., Sherman P., Quesada C., Fawaz M. V., Desmond T. J., Scott P. J. H. (2014). Synthesis
and evaluation of [^11^C]­PyrATP-1, a novel radiotracer for
PET imaging of glycogen
synthase kinase-3β (GSK-3β). Nuclear
Medicine and Biology.

[38] Kumata K., Yui J., Xie L., Zhang Y., Nengaki N., Fujinaga M., Yamasaki T., Shimoda Y., Zhang M.-R. (2015). Radiosynthesis and
preliminary PET evaluation of glycogen synthase kinase 3β (GSK-3β)
inhibitors containing [^11^C]­methylsulfanyl, [^11^C]­methylsulfinyl or [^11^C]­methylsulfonyl groups. Bioorg. Med. Chem. Lett..

[39] Wang M., Gao M., Miller K. D., Sledge G. W., Hutchins G. D., Zheng Q.-H. (2011). The first
synthesis of [^11^C]­SB-216763, a new potential PET agent
for imaging of glycogen synthase kinase-3 (GSK-3). Bioorg. Med. Chem. Lett..

[40] Li L., Shao X., Cole E. L., Ohnmacht S. A., Ferrari V., Hong Y. T., Williamson D. J., Fryer T. D., Quesada C. A., Sherman P., Riss P. J., Scott P. J. H., Aigbirhio F. I. (2015). Synthesis
and Initial in Vivo Studies with [^11^C]­SB-216763: The First
Radiolabeled Brain Penetrative Inhibitor of GSK-3. ACS Med. Chem. Lett..

[41] Hu K., Patnaik D., Collier T. L., Lee K. N., Gao H., Swoyer M. R., Rotstein B. H., Krishnan H. S., Liang S. H., Wang J., Yan Z., Hooker J. M., Vasdev N., Haggarty S. J., Ngai M.-Y. (2017). Development
of [^18^F]­Maleimide-Based
Glycogen Synthase Kinase-3β Ligands for Positron Emission Tomography
Imaging. ACS Med. Chem. Lett..

[42] Liang S. H., Chen J. M., Normandin M. D., Chang J. S., Chang G. C., Taylor C. K., Trapa P., Plummer M. S., Para K. S., Conn E. L., Lopresti-Morrow L., Lanyon L. F., Cook J. M., Richter K. E. G., Nolan C. E., Schachter J. B., Janat F., Che Y., Shanmugasundaram V., Lefker B. A., Enerson B. E., Livni E., Wang L., Guehl N. J., Patnaik D., Wagner F. F., Perlis R., Holson E. B., Haggarty S. J., El Fakhri G., Kurumbail R. G., Vasdev N. (2016). Discovery of a Highly Selective Glycogen
Synthase Kinase-3 Inhibitor (PF-04802367) That Modulates Tau Phosphorylation
in the Brain: Translation for PET Neuroimaging. Angew. Chem., Int. Ed..

[43] Prabhakaran J., Zanderigo F., Sai K. K. S., Rubin-Falcone H., Jorgensen M. J., Kaplan J. R., Mintz A., Mann J. J., Kumar J. S. D. (2017). Radiosynthesis
and in Vivo Evaluation of [^11^C]­A1070722, a High Affinity
GSK-3 PET Tracer in Primate Brain. ACS Chem.
Neurosci..

[44] Prabhakaran J., Sai K. K. S., Sattiraju A., Mintz A., Mann J. J., Kumar J. S. D. (2019). Radiosynthesis
and evaluation of [^11^C]­CMP,
a high affinity GSK3 ligand. Bioorg. Med. Chem.
Lett..

[45] Zhong Y., Yang S., Cui J., Wang J., Li L., Chen Y., Chen J., Feng P., Huang S., Li H., Han Y., Tang G., Hu K. (2021). Novel 18F-Labeled Isonicotinamide-Based
Radioligands for Positron Emission Tomography Imaging of Glycogen
Synthase Kinase-3β. Mol. Pharmaceutics.

[46] Gundam S. R., Bansal A., Kethamreddy M., Ghatamaneni S., Lowe V. J., Murray M. E., Pandey M. K. (2024). Synthesis and preliminary
evaluation of novel PET probes for GSK-3 imaging. Sci. Rep..

[47] Xiao Z., Li Y., Haider A., Pfister S. K., Rong J., Chen J., Zhao C., Zhou X., Song Z., Gao Y., Patel J. S., Collier T. L., Ran C., Zhai C., Yuan H., Liang S. H. (2024). Radiosynthesis and evaluation of
a novel ^18^F-labeled tracer for PET imaging of glycogen
synthase kinase 3. Am. J. Nucl. Med. Mol. Imaging.

[48] Wagman A.
S., Boyce R. S., Brown S. P., Fang E., Goff D., Jansen J. M., Le V. P., Levine B. H., Ng S. C., Ni Z.-J., Nuss J. M., Pfister K. B., Ramurthy S., Renhowe P. A., Ring D. B., Shu W., Subramanian S., Zhou X. A., Shafer C. M., Harrison S. D., Johnson K. W., Bussiere D. E. (2017). Synthesis, Binding Mode, and Antihyperglycemic Activity
of Potent and Selective (5-Imidazol-2-yl-4-phenylpyrimidin-2-yl)­[2-(2-pyridylamino)­ethyl]­amine
Inhibitors of Glycogen Synthase Kinase 3. J.
Med. Chem..

[49] Heider F., Pantsar T., Kudolo M., Ansideri F., De Simone A., Pruccoli L., Schneider T., Goettert M. I., Tarozzi A., Andrisano V., Laufer S. A., Koch P. (2019). Pyridinylimidazoles
as GSK3β Inhibitors: The Impact of Tautomerism on Compound Activity
via Water Networks. ACS Med. Chem. Lett..

[50] Van
de Bittner G. C., Ricq E. L., Hooker J. M. (2014). A Philosophy for
CNS Radiotracer Design. Acc. Chem. Res..

[51] Stéen E. J. L., Vugts D. J., Windhorst A. D. (2022). The Application of in silico Methods
for Prediction of Blood-Brain Barrier Permeability of Small Molecule
PET Tracers. Front. Nucl. Med..

[52] Wager T. T., Hou X., Verhoest P. R., Villalobos A. (2010). Moving beyond Rules: The Development
of a Central Nervous System Multiparameter Optimization (CNS MPO)
Approach To Enable Alignment of Druglike Properties. ACS Chem. Neurosci..

[53] Abbott N. J. (2004). Prediction
of blood–brain barrier permeation in drug discovery from in
vivo, in vitro and in silico models. Drug Discovery
Today: Technologies.

[54] Lapchak P. A. (2013). Drug-Like
Property Profiling of Novel Neuroprotective Compounds to Treat Acute
Ischemic Stroke: Guidelines to Develop Pleiotropic Molecules. Translational Stroke Research.

[55] Yao H.-B., Shaw P.-C., Wong C.-C., Wan D. C.-C. (2002). Expression of
glycogen synthase kinase-3 isoforms in mouse tissues and their transcription
in the brain. Journal of Chemical Neuroanatomy.

[56] Leroy K., Brion J.-P. (1999). Developmental expression and localization of glycogen
synthase kinase-3β in rat brain. Journal
of Chemical Neuroanatomy.

[57] Andersson, L. ; Arzel, E. ; Berg, S. ; Burrows, J. ; Hellberg, S. ; Huerta, F. ; Pedersen, T. ; Rein, T. ; Rotticci, D. ; Staaf, K. , New pyrimidine derivatives and their use in therapy as well as the use of pyrimidine derivatives in the manufacture of a medicament for prevention and/or treatment of alzheimer’s disease. WO2007040440A1: 2007.

[58] Dahl K., Itsenko O., Rahman O., Ulin J., Sjöberg C.-O., Sandblom P., Larsson L.-A., Schou M., Halldin C. (2015). An evaluation
of a high-pressure ^11^CO carbonylation apparatus. Journal of Labelled Compounds and Radiopharmaceuticals.

[59] Bhat R., Xue Y., Berg S., Hellberg S., Ormö M., Nilsson Y., Radesäter A.-C., Jerning E., Markgren P.-O., Borgegård T., Nylöf M., Giménez-Cassina A., Hernández F., Lucas J. J., Díaz-Nido J., Avila J. (2003). Structural Insights
and Biological Effects of Glycogen Synthase Kinase
3-specific Inhibitor AR-A014418. J. Biol. Chem..

[60] Bernard-Gauthier V., Mossine A. V., Knight A., Patnaik D., Zhao W.-N., Cheng C., Krishnan H. S., Xuan L. L., Chindavong P. S., Reis S. A., Chen J. M., Shao X., Stauff J., Arteaga J., Sherman P., Salem N., Bonsall D., Amaral B., Varlow C., Wells L., Martarello L., Patel S., Liang S. H., Kurumbail R. G., Haggarty S. J., Scott P. J. H., Vasdev N. (2019). Structural Basis for
Achieving GSK-3β Inhibition with High Potency, Selectivity,
and Brain Exposure for Positron Emission Tomography Imaging and Drug
Discovery. J. Med. Chem..

