# Supplementary material for: Radiosynthesis and Evaluation of 11C‑Labeled Imidazolyl Pyrimidine Derivatives for Positron Emission Tomography Imaging of Glycogen Synthase Kinase‑3
Source: ACS Pharmacol Transl Sci. 2025 Jun 20;8(7):1986–95. doi: 10.1021/acsptsci.5c00032 (PMC12260939; doi:10.1021/acsptsci.5c00032)
Supplement: Supplementary file 1 [file pt5c00032_si_001.pdf]

Supporting Information

Radiosynthesis and Evaluation of ^{11}C-labeled Imidazolyl Pyrimidine Derivatives for Positron Emission Tomography Imaging of Glycogen Synthase Kinase-3

Yinlong Li,[1] Kenneth Dahl,[2,3] Charles S. Elmore,[4] Johan Sandell,[4] Akihiro Takano,[3] Christer Halldin,[3,5] Lars Farde,[3] Charlotte Ahlgren,[3] Alison Cochrane,[4] Jian Rong,[1] Jiahui Chen,[1] Chunyu Zhao,[1] Xin Zhou,[1] Jimmy S. Patel,[1,6] Zhendong Song,[1] Ahmad Chaudhary,[1] Yabiao Gao,[1] Zhenkun Sun,[7] Zachary Zhang,[1] Siyan Feng,[1] Achi Haider,[1] Steven H. Liang[1,]* and Magnus Schou[2,3,]*

[1]Department of Radiology and Imaging Sciences, Emory University, 1364 Clifton Road, Atlanta, Georgia 30322, United States

[2]PET Science Centre, Precision Medicine and Biosamples, Oncology R&D, AstraZeneca, Karolinska Institutet, Stockholm, 17176, Sweden

[3]Department of Clinical Neuroscience, Centre for Psychiatry Research, Karolinska Institutet and Stockholm County Council, Stockholm, 17176, Sweden

[4]Early Chemical Development, Pharmaceutical Sciences, R&D, AstraZeneca Pharmaceuticals, Gothenburg 43183, Sweden

[5]Semmelweis University, Department of Biophysics and Radiation Biology, and HUN-REN TKI, 1094 Budapest, Hungary

[6]Department of Radiation Oncology, Winship Cancer Institute of Emory University, Atlanta, Georgia, 30322, United States

[7]Department of Pharmacology and Chemical Biology, Emory University School of Medicine, Atlanta, Georgia, 30322, United States

Corresponding Authors

Steven Liang − *Department of Radiology and Imaging Sciences, Emory University, 1364 Clifton Road, Atlanta, Georgia 30322, United States*. Email: steven.liang@emory.edu.

Magnus Schou − *PET Science Centre, Precision Medicine and Biosamples, Oncology R&D, AstraZeneca, Karolinska Institutet, Stockholm, 17176, Sweden; Department of Clinical*

Neuroscience, Centre for Psychiatry Research, Karolinska Institutet and Stockholm County Council, Stockholm, 17176, Sweden. Email: magnus.schou@astrazeneca.com.

Content

1) Synthesis of [^{3}H]**13a**, [^{3}H]**13b**, [^{3}H]**13c**, **[^{3}H13d,** and [^{3}H]**13e**
2) TACs of [^{11}C]**13a**, [^{11}C]**13b**, [^{11}C]**13c**, [^{11}C]**13d** and [^{11}C]**13e** in various NHP brain regions

General Information

The syntheses of [^{3}H]**13a**, [^{3}H]**13b**, [^{3}H]**13c**, [^{3}H]**13d**, and [^{3}H]**13e** were achieved by a common process of iodination of the unlabeled compound on the benzene ring using *N*-iodosuccinimmide in trifluoroacetic acid. The tritium-labeled compound was then produced by tritio-dehalogenation using T_2 and PdO. Preparative HPLC was conducted on a 10 x 250 mm or 19 x 250 mm Waters Xbridge C-18 or C-8 column, using 40-80% CH_3CN-50mM NH_4OAc. Analytical HPLC was performed on a 2.1 x 100 mm Zorbax SB C-18 or Waters Xbridge C-18 or 3.0 x 10 mm Atlantis C-18 column using gradient elution from 5 to 95% of MeCN - 95% 10 mM NH_4OAc - 5% MeCN over 15 min. LCMS was performed on a 3.0 x 50 mm Waters Xbridge C-18 column using gradient elution from 5 to 95% of MeCN - 95% 10 mM NH_3 (adjusted to pH 10 with HOAc) over 5 min.

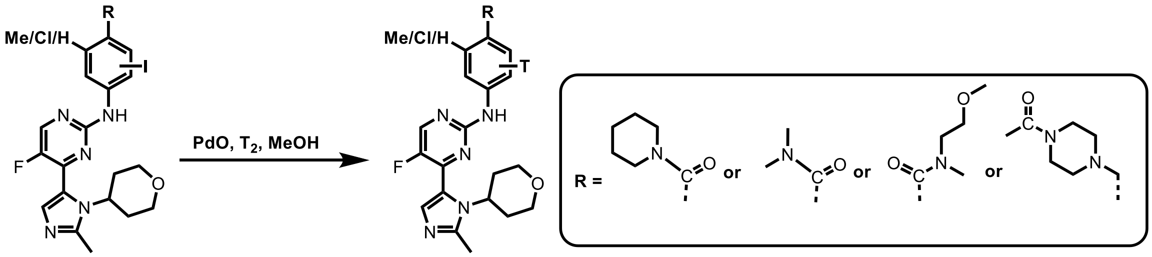

17a-e

[^{3}H]13a-e

Scheme S1. Syntheses of [^{3}H]**13a-e.**

*Synthesis of [^{3}H]**13a** ([^{3}H]AZ12646326)*

A slurry of 1.0 mg (1.6 µmol) of (2-chloro-4-(5-fluoro-4-(2-methyl-1-(tetrahydro-2H-pyran-4-yl)-1H-imidazol-5-yl)pyrimidin-2-ylamino)-5-iodophenyl)(piperidin-1-yl)methanone and 0.3 mg (2.45 µmol)) of palladium(II) oxide in 0.3 mL of methanol was stirred under a tritium atm at 820 mbar for 1 hour. The catalyst was removed by filtration and the solvent evaporated. The residue was purified by preparative HPLC. The product-containing fractions were combined, and the solvent evaporated. The residue was taken up in 5 ml of ethanol to give 97.5 MBq. The radiochemical purity was determined to be > 99.5% with a UV area % (254 nm) of 77%. The molar activity was determined to be 732 GBq/mmol. LC/MS: 498.9 (37.9%), 500.8 (100%), 502.0 (9.0%), 502.8 (13.0%).

*Synthesis of [^{3}H]**13b** ([^{3}H]AZ12646603)*

The tritiodehalogenation was conducted using 1.0 mg of 2-chloro-4-(5-fluoro-4-(2-methyl-1-(tetrahydro-2H-pyran-4-yl)-1H-imidazol-5-yl)pyrimidin-2-ylamino)-5-iodo-*N,N*-dimethylbenzamide and 750 mBar pressure of tritium gas to afford 137 MBq of [^{3}H]**13b**. The radiochemical purity was determined to be 99% with a UV area % (254 nm) of 99%. The molar activity was 652 GBq/mmol. LCMS: 459.2 (44.4%), 461.2 (100%), 463.2 (32.0%).

*Synthesis of [^{3}H]**13c** ([^{3}H]AZ12656261)*

The tritiodehalogenation was conducted using 4.1 mg of 4-(5-fluoro-4-(2-methyl-1-(tetrahydro-2H-pyran-4-yl)-1H-imidazol-5-yl)pyrimidin-2-ylamino)-5-iodo-*N,N*,2-trimethylbenzamide and 815 mBar pressure of tritium gas to afford 63 MBq of [^{3}H]AZ12656261. The radiochemical purity was determined to be >99.5% with a UV area % (285 nm) of >99%. The molar activity was determined to be 704 GBq/mmol. LCMS 439.2 (48.6), 441.2 (100%), 443.2 (7.1%).

*Synthesis of [^{3}H]**13d** ([^{3}H]AZ12977360)*

The tritiodehalogenation was conducted using 1.0 mg of 4-(5-fluoro-4-(2-methyl-1-(tetrahydro-2H-pyran-4-yl)-1H-imidazol-5-yl)pyrimidin-2-ylamino)-3,5-diiodo-*N*-(2-methoxyethyl)-*N*,2-dimethylbenzamide and 1086 mBar pressure of tritium gas to afford 80 MBq of [^{3}H] AZ12977360. The radiochemical purity was determined to be 98% with a UV area % (254 nm) of 92%. The molar activity was determined to be 632 GBq/mmol. LCMS: 483.3 (90.4), 485.2 (100%), 487.2 (16.6%).

*Synthesis of [^{3}H]**13e** ([^{3}H]AZ12943203)*

The tritiodehalogenation was conducted using 1.1 mg of 1-(4-(4-((5-fluoro-4-(2-methyl-1-(tetrahydro-2H-pyran-4-yl)-1H-imidazol-5-yl)pyrimidin-2-yl)amino)-3-iodobenzyl)piperazin-1-yl)ethan-1-one and 683 mBar pressure of tritium gas to afford 527 MBq of [^{3}H]AZ12943203. The radiochemical purity was determined to be >99.5% with a UV area % of 98%. The molar activity was determined to be 750 GBq/mmol. LCMS: 494.4 (29.8%), 496.4 (100%), 498.4 (5.4%).

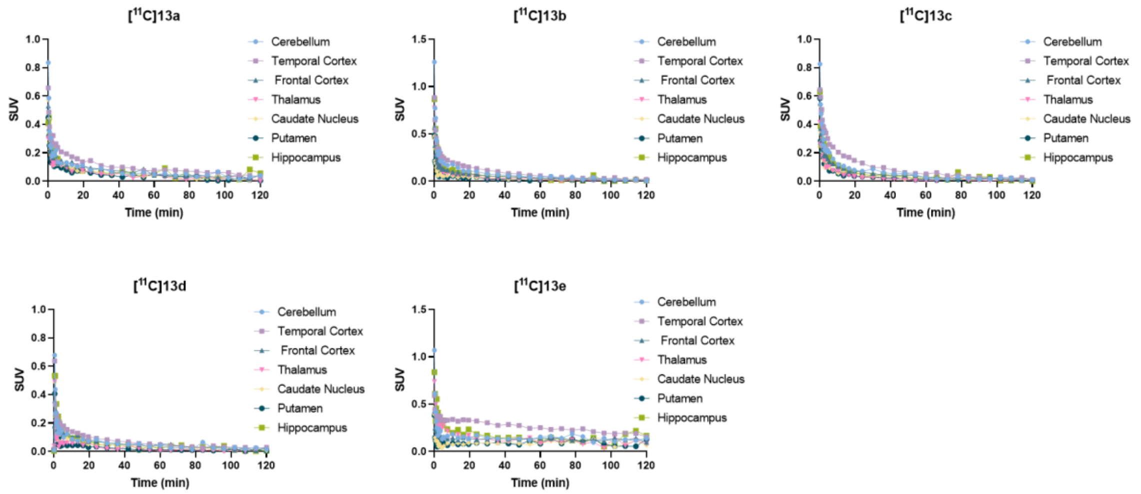

Figure S1. TACs of [^{11}C]**13a**, [^{11}C]**13b**, [^{11}C]**13c**, [^{11}C]**13d** and [^{11}C]**13e** in various NHP brain regions.
